# Recent advances in targeted drug delivery systems for resistant colorectal cancer

**DOI:** 10.1186/s12935-022-02605-y

**Published:** 2022-05-19

**Authors:** Masoumeh Sharifi-Azad, Marziyeh Fathi, William C. Cho, Abolfazl Barzegari, Hamed Dadashi, Mehdi Dadashpour, Rana Jahanban-Esfahlan

**Affiliations:** 1 Department of Medical Biotechnology, Faculty of Advanced Medical Sciences, Tabriz University of Medical Sciences, Tabriz, Iran; 2 Research Center for Pharmaceutical Nanotechnology, Biomedicine Institute, Tabriz University of Medical Sciences, Tabriz, Iran; 3 Department of Clinical Oncology, Queen Elizabeth Hospital, Hong Kong SAR, China; 4 Department of Medical Biotechnology, Faculty of Medicine, Semnan University of Medical Sciences, Semnan, Iran; 5 Cancer Research Center, Semnan University of Medical Sciences, Semnan, Iran

**Keywords:** Colorectal cancer, Drug delivery, Single cell approaches, Artificial intelligence, Exosome, Circulating tumor cells, Cancer stem cells

## Abstract

Colorectal cancer (CRC) is one of the deadliest cancers in the world, the incidences and morality rate are rising and poses an important threat to the public health. It is known that multiple drug resistance (MDR) is one of the major obstacles in CRC treatment. Tumor microenvironment plus genomic instability, tumor derived exosomes (TDE), cancer stem cells (CSCs), circulating tumor cells (CTCs), cell-free DNA (cfDNA), as well as cellular signaling pathways are important issues regarding resistance. Since non-targeted therapy causes toxicity, diverse side effects, and undesired efficacy, targeted therapy with contribution of various carriers has been developed to address the mentioned shortcomings. In this paper the underlying causes of MDR and then various targeting strategies including exosomes, liposomes, hydrogels, cell-based carriers and theranostics which are utilized to overcome therapeutic resistance will be described. We also discuss implication of emerging approaches involving single cell approaches and computer-aided drug delivery with high potential for meeting CRC medical needs.

## Introduction

**Fig. 1:**
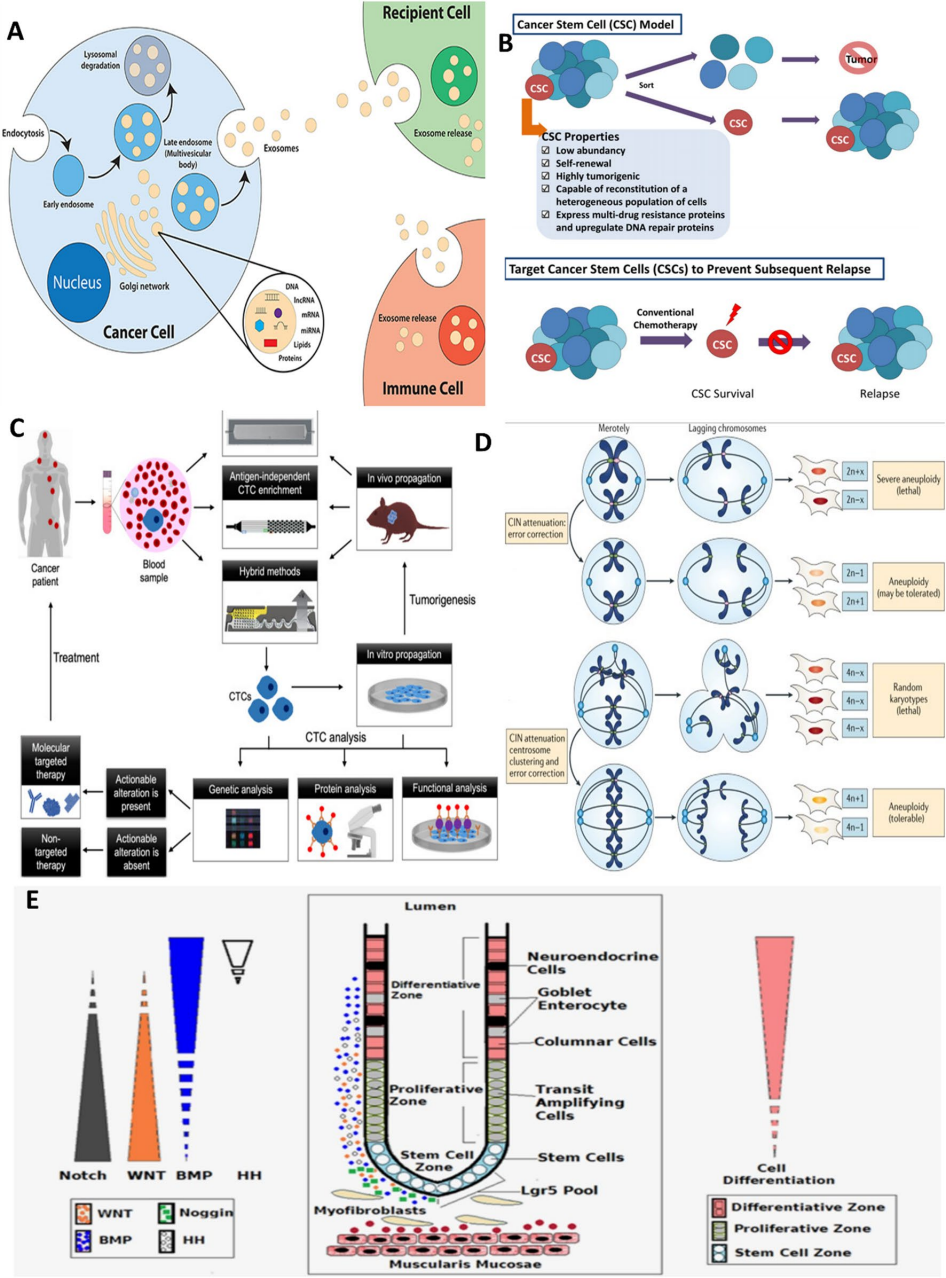
Representation of different drug resistance mechanisms in CRC.** a** cancer-derived exosome. Reprinted from [24] under Creative Commons Attribution License.4.0. Copyright (2019) Frontiers. **b** Cancer stem cells. Reprinted from [25] with permission, Copyright (2018) John Wiley and Sons. **c** Circulating tumor cells. Reprinted from [26] under Creative Commons Attribution License.4.0. Copyright (2021) John Wiley and Sons. **d** Genomic instability. Reprinted from [27] with permission, Copyright (2018) Springer Nature. **e** Signaling pathways. Reprinted from [28] with permission, Copyright (2015) John Wiley and Sons

CRC is second most detected health condition and fourth most common fatal cancer around the world [1]. Etiology of CRC is shown to be related to some factors such as age, gender (male), genetic and colorectal cancer syndromes, ethnicity (American, African), obesity, other colon conditions and nutrition (overconsuming of red meat) [2]. Traditional cancer therapy including surgery, radiation, cryosurgery and chemotherapy are prone to exhibit undesired side effects and toxicity for patients [3]. Multiple drug resistance (MDR) can be considered as an obstacle for controlling disease and inhibition of tumors [4]. Different factors cause MDR like overexpression of ABC transporter especially P-gp, mutations [5], DNA damage and resistance to former chemotherapy drug due to clonal evolution, tumor heterogeneity [6], presence of cancer stem cell-like cells [7], dormancy [8], hypoxia [9], immune evasion [10], horizontally gene/material transfer [11], and tumor microenvironment complexity [12], among others. In this era, targeted drug delivery has been developed to reduce systematic toxicity and unwanted effects. Nano targeted drug delivery systems (NTDDs) have become popular among researchers to manage cancers [13]. Different strategies can be used to design bioresponsive nanoparticles (NPs) [14] including pH-dependent [15], thermal [16], enzyme [17], redox sensitive [18], ligand-based [16] and magnetically driven systems [19]. In addition, there are also different nanovectors such as liposome, dendrimers, carbon nanotubes, PEG polymers and others that deliver therapeutic agents to tumor sites [20, 21]. These NPs should be designed compatible and responsive to colon site [22]. Main characteristics of colon cancer microenvironment include excessive ROS contents, higher pH than upper GI tract, different enzyme activity, inflammation and others [23]. Herein, first the underlying causes of MDR will be introduced (Fig. 1), following recent achievements and future prospective around NTDDs for treatment of resistant CRC will be discussed.

## Drug resistance mechanisms in CRC

### Tumor derived exosome (TDE)

Exosomes are plasma membrane-driven vesicles that shed from different cells and can be detected in body fluids. In cancer cells, these vesicles transfer genetic materials, as well as proteins to distant sites leading to tumor progression, metastasis and drug resistance [24]. Angiogenesis is a process which forms new vasculature and is induced by vascular endothelial growth factor (VEGF), fibroblast growth factor (FGF), platelet-derived growth factor (PDGF), basic fibroblast growth factor (bFGF), transforming growth factor β (TGF-β), tumor necrosis factor α (TNF-α) and interleukin-8 (IL-8) and these factors can be carried by TDEs [12]. Besides, some exosomal-derived miRNAs have been detected that play a role in regulating angiogenic transcription factors, promoting angiogenesis and MDR in CRC [4]. Studies on rich-nutrition wheatgrass juice (WGJ) that extracted from plant Triticum aestivum, revealed post-chemotherapy improvement and modifying tumor associated microvesicles including exosomes properties by daily consumption of WGJ in CRC patient undergoing chemotherapy [29]. Moreover, exosomes containing miR-934 can shift the macrophages into M2 phase and promote liver metastasis of colorectal cancer which is one of the deadliest consequences of CRC [30]. Beside the role of exosomes in CRC metastasis, progression and resistance, they also serve as potential biomarkers for detecting CRC [31].

### Cancer stem cells (CSCs)

One of the major challenges in cancer recovery is remaining CSCs after conventional therapy which regain their renewal and dedifferentiation properties [32]. Plus, tumor dormancy enables tumor cells to be silent but alive and regain proliferation again upon receiving suitable signals resulting in recurrent CRC. Dormancy can be divided into four groups including; primary cancer dormancy, metastatic dormancy, therapy-induced dormancy, immunologic dormancy and their mechanisms are multi-factorial [8]. Autophagy can promote cancer dormancy by keeping them alive and is needed for switching tumor cells to proliferation phase. Different drugs which are used in cancer therapy induce autophagy and thereby lead to resistance. So realizing autophagy mechanism in details to suppress both drug resistance and recurrence requires further investigation [33]. Characterization of CD44/CD133-positive CRC stem cells is highly recommended in order to find novel and effective drugs to treat resistant CRC [34]. For this purpose, some technologies such as whole genome sequencing, single cell approaches and RNA sequencing are promising [35]. By the aid of single cell analysis in CRC, some features like chromosomes copy number variation or shorter telomers in CSCs have been illustrated which were distinct from normal stem cells and cancer epithelial cells [36]. Signaling pathways especially WNT/B-catenin plays vital rules in chemoresistance of colorectal CSCs. Other key pathways attributed to higher maintenance of CSC as well as increased cellular growth, survival, chemoresistance, increased cancer recurrence and metastasis include Notch, Hedgehog, PI3K/AKT, and Hippo/Yap [37]. Likely, overexpression of lipoprotein receptor-related protein 5 (LRP5) in canonical WNT/B-catenin pathway is shown to promote CSC properties in CRC providing a promising target to combat CSC-related resistance [38].

### Circulating tumor cells/DNA

**Fig. 2:**
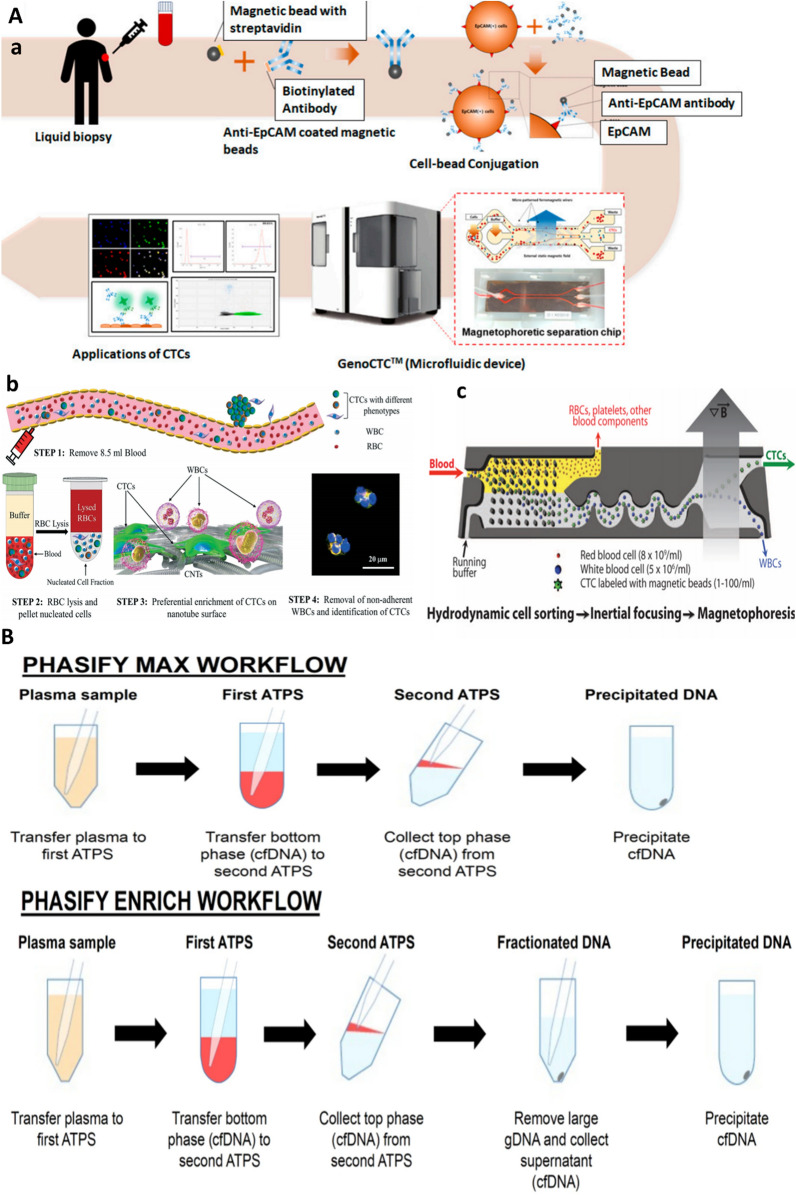
Methods for circulating materials analysis for cancer precision therapy. **A**. CTC enrichment methods. **a** Antigen-dependent (immunoaffinity-based). From [46] under Creative Common Attribution License.3.0. Copyright (2020) MDPI, **b** Antigen-independent methods (e.g. density and size based). Preferential attachment of CTCs on a nanotube chip. Adopted from [47] under Creative Common Attribution License.3.0. Copyright (2019) RSC. **c** Combination approach. Inertial microfluidic focusing for tumor antigen-dependent and independent capture of CTC. Adopted with permission from [48] Copyright (2013) AAAS. **B** Schematic of DNA isolation process with PHASIFY MAX and PHASIFY ENRICH. The PHASIFY method uses serial two-phase liquid extraction systems to isolate and purify cfDNA from a starting plasma sample. In the first aqueous two-phase systems (ATPSs), DNA partitions to the bottom phase (red), which is then extracted and transferred to a second ATPS. After phase separation, the DNA partitions to the top phase (red), which is then extracted. In the PHASIFY MAX workflow, all extracted DNA undergoes DNA precipitation. In the PHASIFY ENRICH workflow, the extracted DNA is first mixed with a fractionation solution to remove contaminating DNA and enrich the sample with potential tumor cfDNA. The enriched sample then undergoes DNA precipitation. Reprinted from [40] with permission, Copyright (2021) Springer Nature

Significant correlation between presence of CTCs and tumor resistance with EMT ability has been indicated. Also, these CTCs can gain stem cell features and lead to CRC recurrence and metastasis. Due to low quantity of CTCs which can be obtained by liquid biopsy from patient’s blood samples, enrichment procedures including antigen-dependent CTC enrichment, antigen- independent CTC enrichment and combination of those protocols are required (Fig. 2A). In addition to enrichment strategies, some profiling methods such as genetic analysis of CTCs, protein level analysis and functional analysis of CTCs should be considered [26]. When tumor site is inaccessible, liquid biopsy come in handy as it analysis circulating materials such as CTCs and ctDNA [39]. Common ctDNA liquid-phase extraction methods for detection of CTCs are replaced with novel PHASIFY MAX and PHASIFY ENRICH methods [40] (Fig. 2B).

ctDNA can be subjected to next-generation sequencing (NGS) to determine mutation profile which provide useful insight about different pathways and the resistance acquisition [41]. Likely, genetic and transcriptomics heterogeneity of tumors can be interpreted from single CTC sequencing methods and also these kinds of analysis can determine the efficacy of adjuvant therapy, monitor tumor progression and reveal metastasis mechanism in solid tumors [42]. Equally, CTC detection has prognostic value in CRC, as for localized disease, CTCs enumeration help to stratify patients to adjuvant therapies by identifying cases at a high risk for metastasis. In advanced metastatic CRC, CTCs detection can be used for systemic therapy monitoring, therapy resistance detection and risk assessment improvement [43]. Recently, ctDNA sequencing in CRC patients treated with first-line cetuximab chemotherapy with anti-EGFR is used to determine mutational status with clinical outcome in metastatic CRC. Data showed that at the time of progressive disease, 54 new mutations including KRAS and MAP2K1 emerged in ctDNA and that at the time of first response evaluation changes in tumor size were significantly correlated with ctDNA mutational status (average variant allele frequency (AVF)) in plasma [44]. Likewise, serial analysis of ctDNA in stage III CRC involving 169 patients has paved the way beyond detection of minimal residual disease and towards assessing efficacy of adjuvant chemotherapy, ctDNA growth rates and detection of early relapse, where ctDNA detection was served as a strong recurrence predictor post-surgery [45].

### Genomic instability

Whole genome sequencing studies revealed high tumoral genetic heterogeneity which leads to resistance to therapy, recurrence and poor prognosis. Chromosomal instability (CIN) is common form of instability present in tumors including CRC [49]. Different mechanisms such as DNA repair gene defect, TP53 mutation, AURKA and GINS1 high expression [50] can cause CIN and subsequently cancer. Aneuploidy is also common feature of cancers and copy number alteration (CNA) studies on organoid culture revealed de novo emergence of whole-chromosome and sub-chromosomal changes during tumor growth with chromatin errors acting as underlying reasons; as chromatin bridge led to subchromosomal CNAs while, lagging chromatin result in whole chromosomal CNAs. Multipolar spindle defect and acentric chromosome fragment replication are other karyotype alteration reasons [51]. Indeed chromosomal instability result in cancer cell population diversity and thereafter immune escape, inflammation and is a negative indicator for survival rate of cancers [27]. Aneuoploidy as a type of CIN can either induce or suppress tumor formation but in case of CRC aneuploidy and loss of function mutation of APC genes which is part of WNT pathway element are common observations [52]. Retrotransposones such as Long Interspersed Nuclear Elements (LINE) and Short Interspersed Nuclear Elements (SINE) can be inserted elsewhere in entire genome and induce CpG island methylation and is responsible for initiating approximately 1% of CRC [53].

An important chromosomal aberration with clinical significance is microsatellite instability (MSI) present in 15–20% of primary CRC. Tumorigenesis can occur through indel mutation of microsatellite, which code tumor suppressor genes. Indeed, mismatch-repair (MMR) deficient tumors show MSI due to their inability to replicate repeated sequences of microsatellites [48]. MSI status provide invaluable information regarding prognosis, detection of Lynch syndrome, adjuvant chemotherapy guiding, and a companion test for checkpoint blockade inhibitors. In this line, one study described a fully automated molecular method, The Idylla™ as a fast and sensitive MSI assay compared to routine methods such as immunohistochemistry to effectively identify MSI in CRC tumor tissues [54]. Likewise, in patient undergoing colon surgery, circulating tumor DNA (ctDNA) appears in post-operative plasma and pose them at a high risk of recurrence. Moreover, in CRC postoperative plasma 70% microsatellite instability (MSI) is detected with tumor-derived mutations vs 33% in microsatellite stability (MSS) cases. MSI CRC (n = 30) showed highly distinct mutational changes in tumor and postoperative plasma compared with MSS CRC (n = 46) as validated by NGS analysis [55].

### Tumor microenvironment and signaling pathways

Tumor microenvironment (TME) of CRC is composed of extracellular matrix (ECM) and cellular components including immune cells, tumor endothelial cells, tumor associated fibroblast and tumor cells. The interaction of cellular and non-cellular elements through various signaling pathways is another important resistance mechanism in CRC [12].

#### WNT/B-Catenin pathway

WNT/B-Catenin pathway plays an important role in embryonic development and proliferation of cells. WNT proteins translated from WNT genes and act as a ligand which interact with frizzled (FZD) receptor and activate intracellular signals. After this, further activation of Disheveled (DVL) protein occurs which subsequently leads to two discrete pathways namely independent (non-canonical) and B-catenin dependent (canonical) signaling [56]. B-catenin translocate to the nucleus and regulates cell cycle and proliferation. Overexpression of WNT leads to tumorigenic activity and eventually CRC [57]. Since dysfunctional WNT signaling lead to resistance to therapy and poor cancer prognosis, its targeting by *e.g.* WNT/FZD antagonist, LRP5/6 inhibitor, DVL inhibitor, Tankyras inhibitor and CK1 agonist is used to combat CRC [58].

It is reported some fibroblast-driven exosomes which carry WNT are related to stem cell properties and as major reprogramming regulators (dedifferentiation), exosomal WNTs can result in high WNT activity and chemotherapy drug resistance in differentiated CRC cells [59]. Likewise, 5-flourouracil (5-FU) as a fundamental chemodrug activates CSCs via p53 mediated WNT/B-catenin pathway and induces stemness properties, tumor recurrence, and drug resistance. This can be overcome using WNT inhibitors and 5-FU as a treatment [60]. KRAS is one of the important oncogenes in CRC. KRAS mutant cells consume glutamine by using glutaminas and SLC25A22. These cells that express mutant KRAS undergo epigenetic alterations, e.g. hypermethylation in specific genes, followed by activation of WNT/B-catenin leading to proliferation, progression and 5-FU resistance in CRC [61].

#### PI3/AKT pathway

Phosphoinositid 3-kinas (PI3K) is a heterodimer enzyme which add phosphate group to phosphatidyl inositol on the plasma membrane and produce phosphatidylinositol (3,4,5)-trisphosphate (PIP3). AKT also known as protein kinas B (PKB) is downstream effector of PI3K and EGFR. Interaction between AKT and PIP3 leads to AKT activation, then other effectors phosphorylation occurs which result in several cellular process such as growth, survival, apoptosis, migration and cancer progression [62, 63]. It is worth to note that signal transduction through EGFR is mediated through two major pathways: the PI3K/AKT/ PTEN/mTOR and, the RAS/RAF/MAPK/ERK [64]. The PI3K/Akt pathway is activated in 60%–70% of CRC and its activity correlates with prognosis in stage II colon cancer [65]. Inhibitors of this pathway are therapeutic targets for CRC, however in resistant forms, mutational and epigenetic analysis could offer better portrait of CRC outcome (see for review [66]).

#### VEGF/VEGFR pathway

VEGFR is a tyrosine kinase receptor that forms dimer upon VEGF binding. Following phosphorylation occurs which activates downstream signaling cascades such as MAPK/ERK, PI3K/AKT, PLC/PKC, resulting in cell proliferation, survival, angiogenesis and cancer progression [67]. As tumor mass need blood supply and nutrition to survive and also metastasis thus vascular targeting combined with other approaches such as chemo by inhibiting e.g. VEGFR pathway seems an efficient method in cancer therapy [68]. For one, as mTOR inhibitor therapy for gastrointestinal malignancy with TFE3 (transcription factor E3) rearrangement was not satisfactory, a combination therapy of Apatinib which is tyrosine kinase inhibitor (TKI) and anti-VEGFR with chemotherapy drug was adopted [69]. Apatinib treatment induce tumor vascular normalization and fix the problems caused by abnormal tumor vessels including hypoxia, acidosis and thus can reverse drug resistance [70]. Apatinib monotherapy for resistance HER-2 positive breast cancer which show no response to multiple HER-2 therapy, is a kind of prospective treatment which obtained partial remission (PR) and significant progression free stage (PFS) [71]. The standard third-line treatment of metastatic CRC includes the anti-vascular small-molecule drugs (regofenib and fruquintinib) and the tipiracil hydrochloride (TAS-102) and trifluridine chemotherapy drugs [72]. This combination therapy is adopted as single anti-angiogenic therapy was faced with resistance and even promoted metastasis [73]. Accordingly, a case study reported successful treatment of a CRC patient with RAS/BRAF wild-type using a combination of anti-VEGF (fruquintinib) and anti-EGFR (cetuximab) drugs for the treatment of previously treated metastatic CRC, after resistance to chemotherapy, cetuximab, bevacizumab, and regorafenib [74].

#### HGF/cMET pathway

HGF (hepatocyte growth factor) is kind of cytokine family and is a specific ligand for c-MET (cellular-mesenchymal–epithelial transition factor), which is a kinase receptor. Upregulation of this pathway is involved in several cancers, including CRC and causes multiple effects including increased proliferation, EMT, invasion, metastasis, drug resistance, and enhanced cancer cell metabolism and biogenesis [75, 76]. Thus, targeted therapy of HGF/cMET pathways are exploited using TKIs such as crizotinib, Cmet or HGF blocking agent such as onartuzumab, emibetuzumab, and JNJ-61186372, which is a bispecific Ab against cMET and EGFR. Similarly LY3164530 target both c-MET and EGFR and show more efficiency than emibetuzumab or drugs such as volitinib, gefitinib, SAR125844 which is triazolopyridazne derivative, Tepotinib, and capmatinib [77, 78]. One of the main causes of mortality in CRC is liver metastasis. In this respect, an increment in CD4^+^ forkhead box p3 (Foxp3)^+^ Tregs (regulatory T cells) and the HGF/c-Met signaling pathway along with upregulation of HGF/c-MET signaling is reported which inhibits cytotoxic T cell and thus metastasis and invasion increases [79]. C-MET expression evaluated in four groups of cells including colon mucosa, primary CRC, liver and CRC metastatic liver. Results indicated that the highest c-MET expression in CRC metastasis liver correlated with disease stages, invasion and poor prognosis [80]. Also, downregulating of HGF/c-MET pathway was promising using miRNAs including MIR-1, MIR34, MIR 141, MIR199, MIR206 [81].

Other data revealed association between CD44 expression and CRC metastasis. This occurs through CRC derived carcinoma-associated fibroblast (CAFs), as one of the components of TME, which up-regulates CD44 by HGF/C-MET pathway and promote adhesion and migration of CRC tumor cells in metastatic animal model by HGF secretion [82].

### Radio resistance

As we know, radiotherapy (RT) is a non-invasive procedure with side effects that can be applied to various types of cancer including CRC. Resistances to RT can occur due to different factors such as induction of EMT, amplified DNA repair, increased telomerase activity and etc. [83]. In one study CRC patients were investigated and JAK2/STAT signaling axis reported as a radio resistant factor by decreasing apoptosis which led to persistent growth of CSCs after RT [84]. Another study revealed the role of long noncoding RNA LINC00630 in promoting CRC radio resistance by epigenetically regulating of BEX1 [85]. Moreover, enhanced expression of FOXQ1 occurs in CRC and its knockdown shows reduced radio resistance due to affecting B-Catenin nuclear translocation and decreasing intestinal pathological bacteria [86].

### Multiple drug resistance (MDR)

MDR is considered as a predominant reason of cancer mortality. Various factors such as promoted efflux of drugs, genetic factors, growth factors, increased metabolism of xenobiotics and incremented DNA repair can lead to MDR [87]. Cellular membrane lipid content in particular; phospholipids and cholesterol are important in MDR. They modulate the expression and activity of efflux pump by four ways including (I) decreased membrane fluidity and change in membrane structure, (II) Lower amount of oxidizable fatty acid, (III) reduced amount of cytotoxic reactive aldehyde in MDR cells and (IV) activating several signaling cascade that lead to MDR by existence of high amount of lipid precursors [88]. p-gp as an ABC membrane transporter is overexpressed in MDR cancer cells. It is found that PI3K subunits, P110a and P110B can be targeted to overcome MDR via downregulating p-gp [89].

## Advanced strategies for CRC drug delivery

**Fig. 3:**
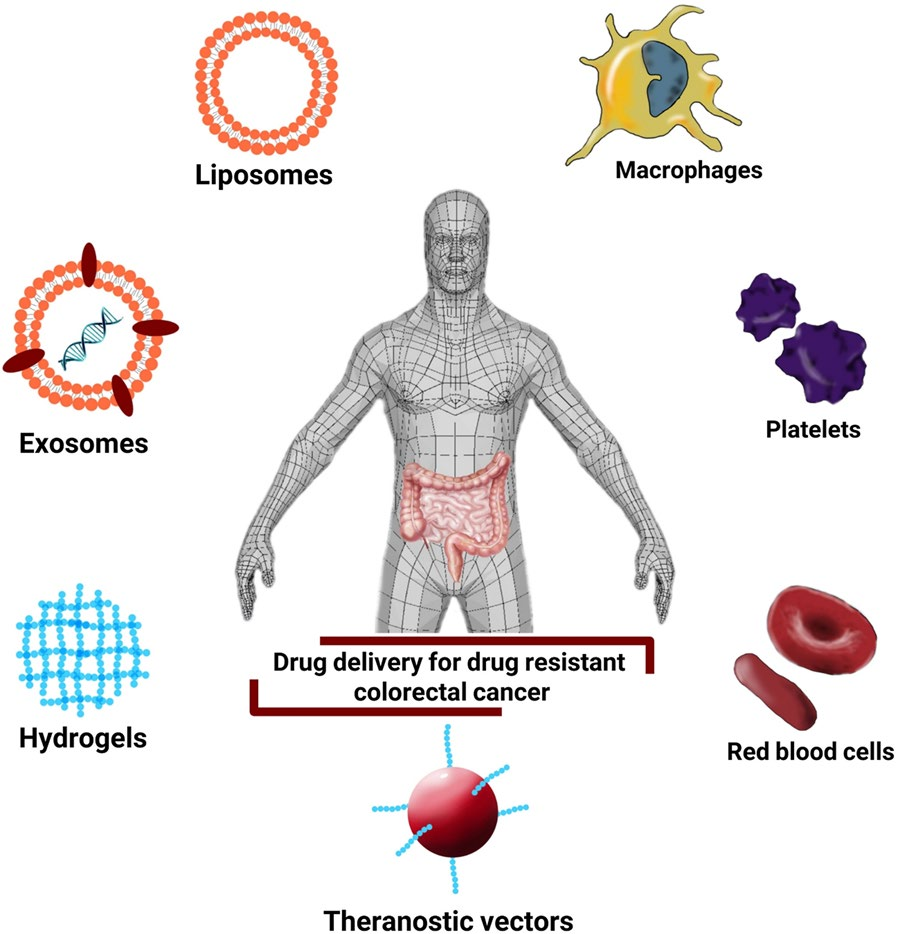
Variety of advanced NTDDs for targeting resistant colorectal cancer

Colon targeting drug delivery systems as an emerging tool has gained popular interest among researchers, since various side effects and low survival of patients arouse from traditional therapies, alternative treatment, in specific targeted drug delivery systems were developed [20, 90]. To achieve the best delivery to CRC, some considerations should be taken into the account, such as CRC microenvironment properties, tumor heterogeneity profile, chemophysical properties of drugs, and colon transient time, among others [91]. Different vectors and strategies can be used as NTDDs (Fig. 3) that we intend to review in detail in following.

### Vectors and vesicles

#### Exosomes

**Fig. 4:**
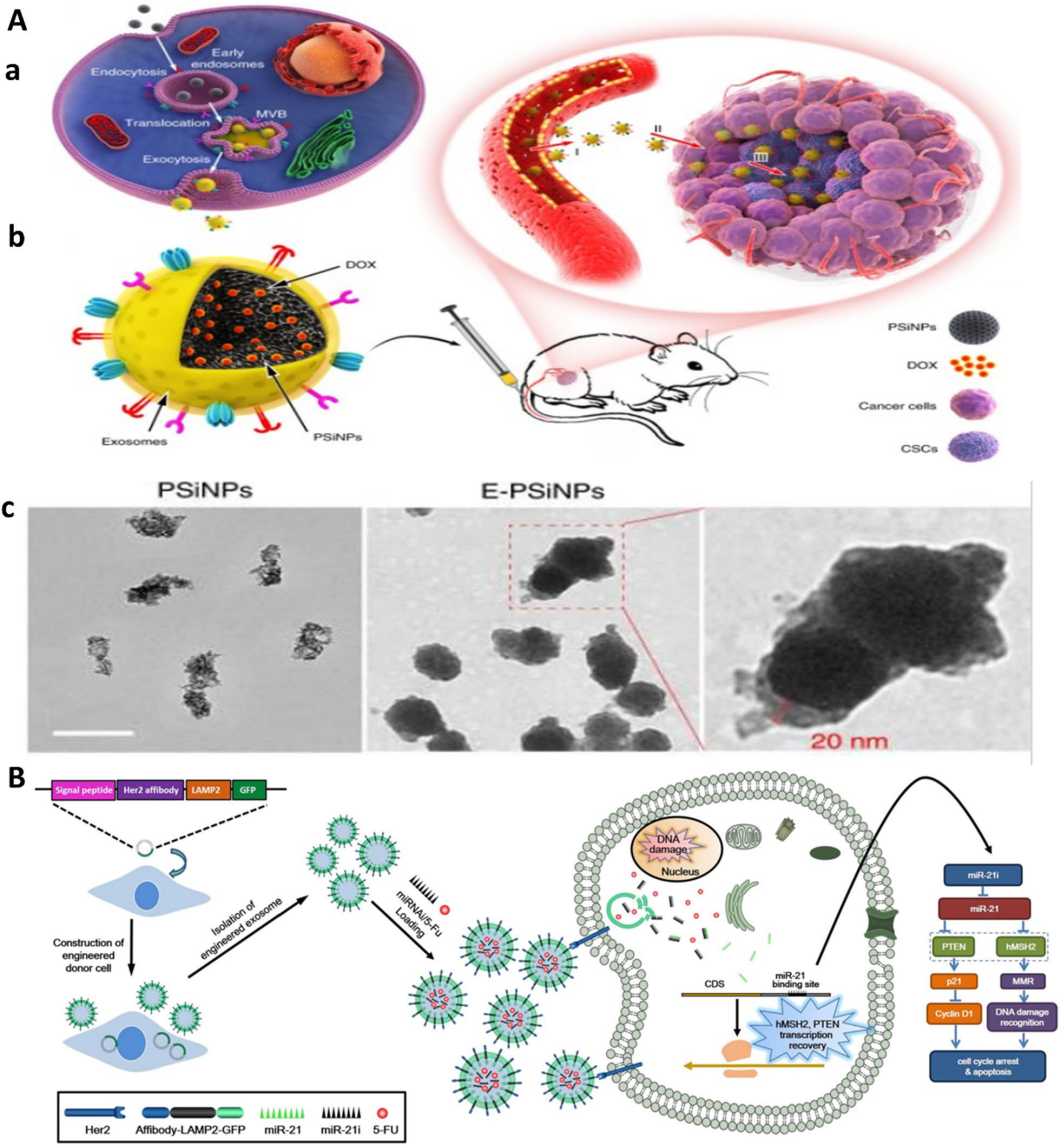
Exosome-based drug carriers for targeted resistant CRC treatment. **A**. **a** Illustration of the preparation of DOX@E-PSiNPs. DOX@PSiNPs are endocytosed into cancer cells after incubation, then localized in MVBs and autophagosomes. After MVBs or amphisomes fuse with cell membrane, DOX@E-PSiNPs are exocytosed into extracellular space. **b** Schematics showing how DOX@E-PSiNPs efficiently target tumor cells after intravenous injection into tumor-bearing mice. (I) DOX@E-PSiNPs efficiently accumulate in tumor tissues; (II) DOX@E-PSiNPs penetrate deeply into tumor parenchyma; and (III) DOX@E-PSiNPs are efficiently internalized into bulk cancer cells and CSCs to produce strong anticancer efficacy. **c** TEM images of PSiNPs and E-PSiNPs. Adopted from [94] under Creative Common Attribution License.4.0. Copyright (2019), Springer Nature. **B** Engineered exosome specifically target CSCs and reverse CRC drug resistance. From [95] with permission. Copyright (2020) Springer Nature

Exosomes are small bilayer structures (40–120 nm) secreted from living cells and can be found in body liquids. As genetic transfer materials, various elements such as DNA, RNA, and protein can be packed and transfer by exosomes among cells. Exosome isolation faces some challenges such as low purity. Among different extraction methods, ultracentrifugation, size-based isolation method, polymer precipitation and immunoaffinity capture techniques are more common. Aside from their prognostic value in CRC and other cancers, as discussed in earlier chapters, several characteristics including endogenous cellular sorting and packaging, inherent capability to cross biological barriers, safety and lack of immunological responses make exosomes as amazing vehicles for targeted drug delivery to cancer [92, 93]. In this respect, CSCs can be targeted by exosome-coated NPs. For one, tumor-cell-exocytosed exosome-biomimetic porous silicon nanoparticles (PSiNPs) loaded with DOX affords superior tumor accumulation, blood vessel extravasation, deep tumor parenchyma penetration, enhanced antitumor and in particular anti-CSCs activity (Fig. 4A) [94]. One of the elements which can be carried by exosomes are miRNAs. Xiao et al. developed engineered exosomes which carry 5-FU and miR-21 inhibitor as a co-delivery system. The engineered exosome contained a Her2-LAMP2 fusion protein which could be expressed on the surface of exosome for specific targeting of Her antigen expressing CRC cells and also their further endocytosis-mediated cellular internalization. The engineered exosome was capable of reversing 5-FU resistance in HCT-1165^FR^ cell line (Fig. 4B) [95]. Moreover, exosomes that secreted from dendritic cells (DC) can pack 5-FU inside themselves and show rebate in CRC cells proliferation, promoting apoptosis, reduction in migration rates and enhancing anti colon cancer effects of 5FU-DC-exo compared with free 5–FU [96]. Different factors including smoking, rich-fat diet and pollution agents cause destructive changes in colon microbiota or enzyme expression that in turn, lead to tumor progression. To address this issue, some researchers have been done. In one study, Gupta et al. reported exosomes extracted from bovine milk (ExoAnthos) by differential centrifugation. And used it for encapsulation of berry-derived anthocyanidins, which has chemoprevention effects on microbiota-driven CRC. This exosomal system enabled higher selectivity of exoanthos activity, reduced tumor mass, normalized enzyme expression and decreased proliferation of tumors in mice and can be effective in prevention and therapy of bacteria-driven colon cancer development [97]. As A33-antigen is expressed highly in CRC cells, therefore Gao et al. used A33-positive LIM1215 cells (A33-Exo) as a resource for extracting exosomes and DOX loading. Following magnetic NPs (US) were prepared displaying A33-antibody on their surface to afford their binding to A33-Exo. Further A33Ab-US-Exo/Dox complex were used to specifically target A33-positive colon cancer cells. This complex showed high tumor cell uptake, increased mice survival rate and reduced cardiotoxicity [98]. Likewsie, mesenchymal stem cell (MSC) driven exosomes loaded with DOX and functionalized with MUC1 (another antigen overexpressed by CRC cells) aptamer on the surface demonstrate high efficacy of targeted drug delivery with lower side effects owing to the enhanced liver clearance of DOX compared to DOX alone [99]. Lan et al. exploited exosome as theranostics for simultaneous CRC therapy and imaging. In this case, exosomes were extracted from tumor cells, and were labeled with radionucleutide and Cy5 to create multimodality imaging probe which target the CRC cells efficiently through single-photon emission computed tomography (SPECT) and near-infrared fluorescence (NIRF) imaging of colon cancer in mice [100]. Not to forget exosomal miRNA applications in diagnosis, prognosis and treatment of CRC. For example, in oxaliplatin–resistance CRC, exosomal delivery of miR-128-3P to CRC cells is shown to promote response to oxaliplatin, reduce EMT, and decline pumping drug outside of tumor cells [101]. Moreover, circ-FBXW7 transferred by exosomes can bind to miR-18b-5p, consequently reverse CRC cells resistance to oxaliplatin, increase apoptosis, and decrease EMT [102].

#### Liposomes

Liposomes are lipid bilayer structures which include both hydrophilic and hydrophobic parts. Because of easy preparation, similarity to membrane and subsequently better cellular uptake, capacity of loading both water soluble and non-soluble drugs due to amphipathic properties, liposomes have gained huge interest as incredible nanodrug carriers for CRC targeting [103]. For example, targeting Urotensin-II receptor (UTR) which is overexpressed in CRC, is reported via liposomes functionalized by UT-II peptides on the surface and loaded with doxorubicin. Such liposomal drug delivery system can specifically target UTR-overexpressing CRC cells, enhance cellular uptake and impart efficient DOX delivery for boosted tumor growth inhibition [104]. In another study, liposome extracted from grapefruit functionalized with DNA aptamer LA1 and loaded with P-gp siRNA was used to inhibit P-gp expression to combat colon cancer MDR in combination with DOX therapy in vitro and in vivo [105]. Also, survivin T34A coding mRNA loaded in liposome-protamine lipoplex (CLPP), which protect mRNA from degrading, exhibited high anti-tumor effect, safety and high delivery capacity. Survivin T34A have been reported as an anti-cancer agent that induces apoptosis, and utilize mRNA instead of DNA which has some benefits including no integrating in to host genome, consistent expression, biodegradability and simpler delivery than DNA ones [106]. As folic acid receptors are abundantly expressed in different cancers, including CRC, Dorkoosh et al. developed folic acid (FA)-conjugated liposome from Dipalmitoylphosphatidlcholine (DPPC) and loaded with 5-FU to impart targeted liposomal-based drug delivery for effective colon cancer treatment [107]. Using phosphatidyle choline (PC) instead of DPPC can even exhibit higher toxicity for colon cancer cells [108]. SN38 is an irinotecan derivative which potentially shows higher effect on CRC cells and inhibits DNA replication and induces apoptosis via blocking DNA topoisomerase I. To tackle its poor solubility and low stability, Ji et al. developed SN38-liposomal vectors which exhibited safety, efficacy and high anti-tumor effect [109]. Likewise, chitosan coated with Docetaxel-loaded liposome is developed to improve drug delivery properties and Docetaxel solubility to promote enhanced therapeutic potential in CRC treatment [110].

As advanced formats of NTDDs, stimuli-responsive liposome are fabricated for controlled drug release. Ogawa et al. found that phthalocyanin derivative, IR700, modified liposomes, triggered by NIR light showed significant anti tumoral effect even under hypoxia condition [111]. Likewise, thermosensitive liposomes encapsulating Mistletoe lectin-1 (ML1)-a ribosome-inactivating protein- have been studied. Upon thermosensitive manner, liposomes permeability increased when exposed to heat, following release of drug occurred and inhibited tumor growth effectively [112]. Statins, as cholesterol lowering drugs, are known as potent anti tumoral agents due to their impact on suppressing isoprenoids generation and the implicated pathways which play rules in cancer angiogenesis, proliferation and invasion. Therefore, Simvastatin loaded in liposome and applied to CRC cell line afforded targeted therapy and exhibited anti-angiogenesis and anti-proliferative potential [113].

#### Hydrogels

**Fig. 5:**
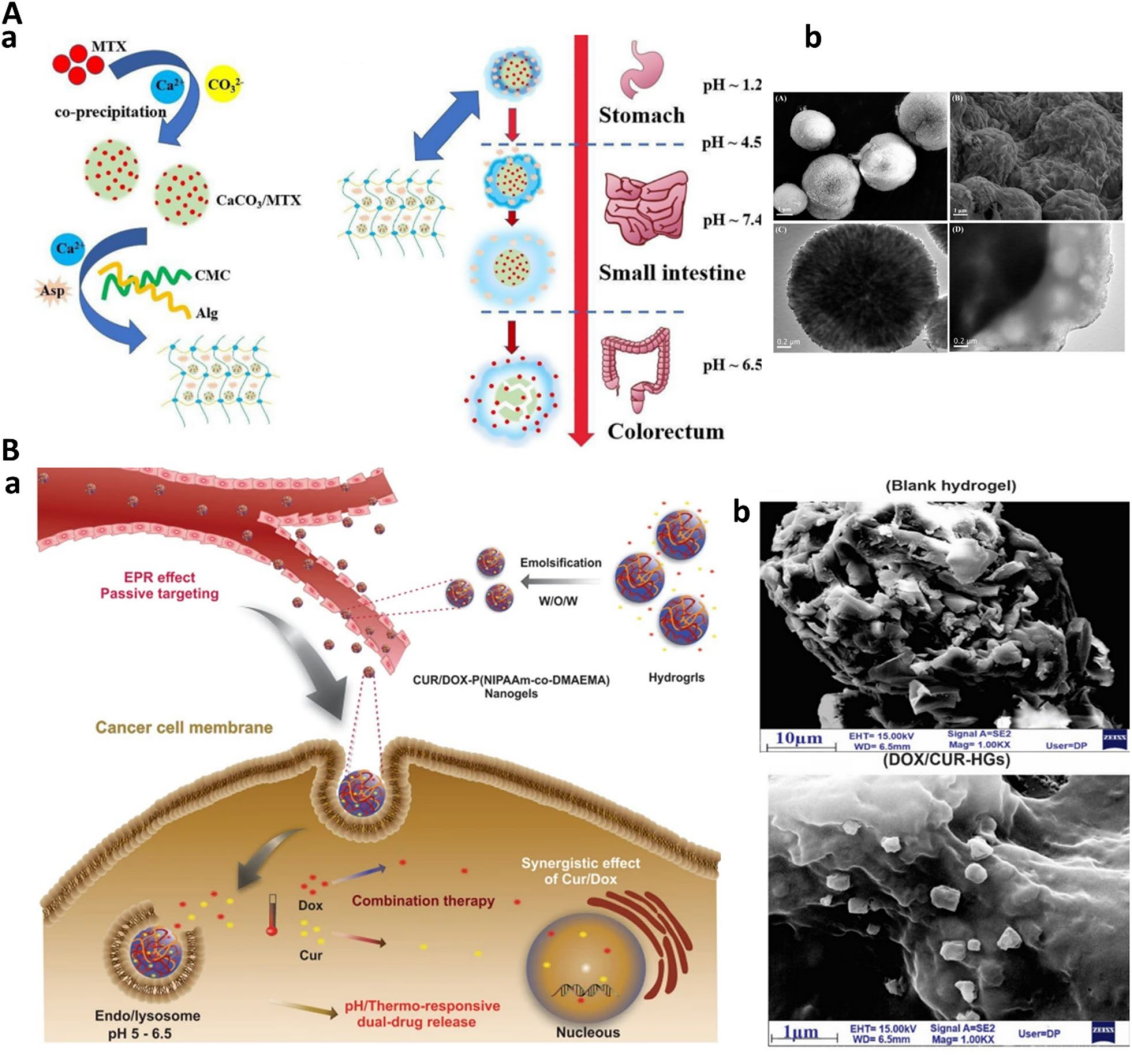
Advanced hydrogel-based drug delivery systems for CRC. **A**. Hybrid hydrogel. Dual hydrogel-based drug delivery system (DDDS). **a** Schematic design and working principle. **b** TEM images of DDDS. From [120] with permission. Copyright (2021) Elsevier. **B. **Bioresponsive hydrogel. pH/thermo-sensitive hydrogels for CRC treatment. a. schematic design of dual-responsive nanogel. b. TEM images of blank nanogel and DOX/CUR- pH thermosensitive nanogel. From [121] under Creative Common Attribution License.4.0. Copyright (2021) Springer Nature

Hydrogels are hydrophilic 3D porous networks, generated through physical or chemical crosslinking of natural or synthetic polymers, in particular polysaccharides. Hydrogel provide a substrate for entrapping different materials with astonishing features namely controlled degradation, sustained cargo release, less toxicity, functionality, and also high capacity of loading drug with transient state formation (sol–gel transition) [114]. Generally, different classification for hydrogels can be provided, for instance, bioresponsive hydrogels [14], bioinspired hydrogels [115], as well as static, dynamic hydrogels, and hybrid ones [116]. Also, different types of hydrogel administration including peroral, rectal, vaginal, ocular, transdermal and implants as same as several therapeutic area like ophthalmic, oral, intestinal, cardiac illness and cancer are also exist [117]. Thermosensitive poloxamer 407 hydrogels which form transient shape from liquid in room temperature to solid in 37 °C, lead to sustained release in body organs and reduce off-target toxicity [118]**.** Different polysaccharides and their composite are used to form hybrid hydrogels including alginate-cyclodextrin, alginate-chitosan, alginate-keratin composite, alginate-PAMAM(G5)hybrid nanogel, and alginate/liposomes hydrogels [119]**.** In this regard, an advanced hybrid dual-drug delivery system (DDDs) involving Ca^+2^ crosslinked hydrogels of alginate and sodium carboxymethyl cellulose is developed. Taking advantage of different pH in small intestine and colorectum, this dual drug delivery system was capable of selective delivery of aspirin and methotrexate-loaded CaCO3 microshperes to their respective target organs [120] (Fig. 5A).Given TME feature of CRC such as high temperature and acidic pH, dual bioresponsvie pH/thermo-sensitive hydrogels loaded with DOX and curcumin (Fig. 5B) prepared by free radical polymerization methods which showed enhanced loading capacity, efficient drug release and induced apoptosis in colon cancer cells [121].Creating hyaluronic acid (HA) and methylcellulose (MC) hydrogels due to thermosensitive feature of MC and intrinsic nature of HA as glycosaminoglycan, made these hydrogels compatible and efficient drug delivery carriers for rectal delivery [122].

#### Bioinics

**Fig. 6:**
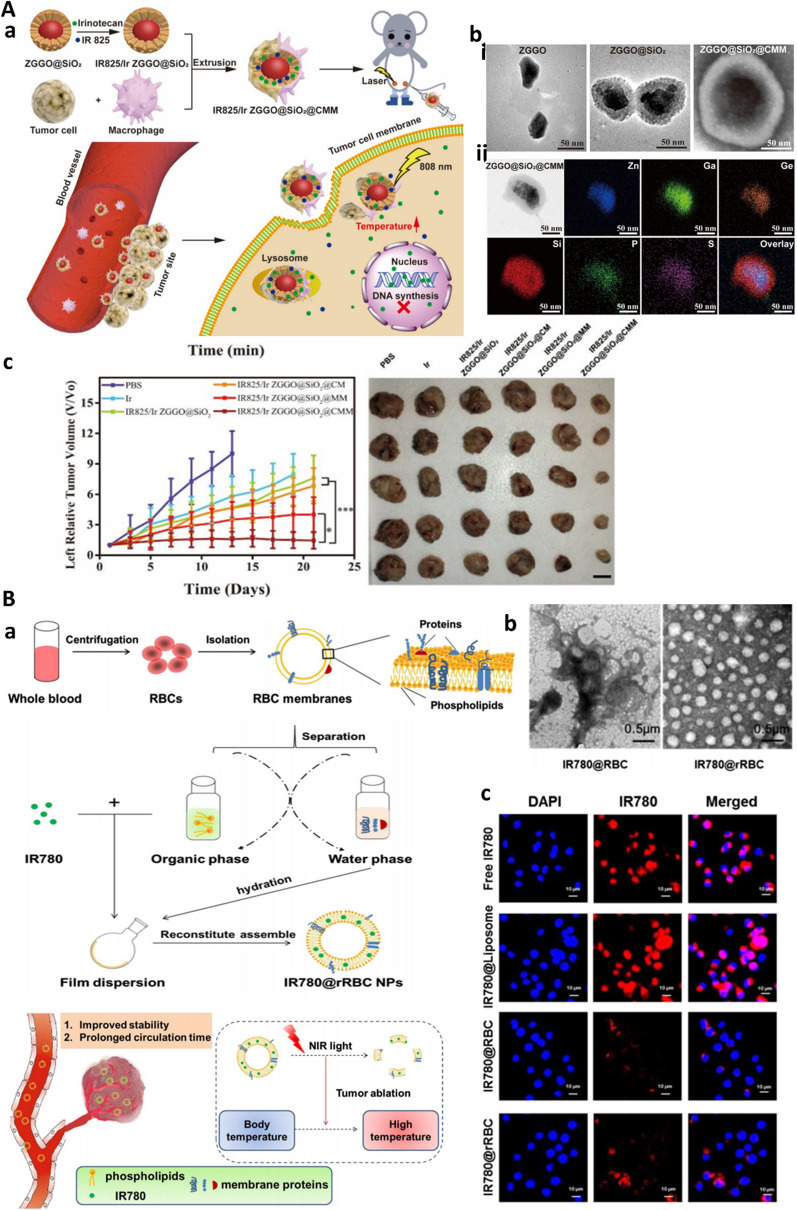
Bionic-based drug delivery to CRC affords immune-cell evasion for efficient NP penetration into CRC microenvironment.** A** Macrophage based drug delivery. **a**, Schematics of cancer cell-macrophage hybrid membrane camouflaged IR825/Ir ZGGO@SiO2@CMM nanoplatform for resistant CRC therapy. **b** TEM images of (i) developed nanomaterials and (ii) element mapping images, **c** The antitumor effect of combined chemo- and photothermal therapy using hybrid camouflaged system on CT26 cancer in mice. Reproduced from [127] with permission. Copyright (2020) American Chemical Society. **B** Biomimetic recombinant red blood cell membranes for improved photothermal therapy of colon cancer. **a** The scheme and procedure for the preparation of IR780 loaded reconstitute RBC membrane nanoparticles (IR780@rRBC NPs). **b** TEM images of IR780@rRBC NPs. **c** In vivo cellular uptake and immune-cell escape potential of RBC-stealth NPs on Raw264.7 macrophage cells. Reprinted with permission from [130] under Creative Common Attribution License.4.0. Copyright (2021), Springer Nature

As conventional target therapy showed some defects like short half time and toxicity, novel cell-based carriers with interesting features such as sustained blood release, low-rate clearance, efficient targeting and biocompatibility became popular. Besides exosomes, discussed in earlier chapters, red blood cells (RBCs), macrophages, platelets (PLTs), neutrophils, bacteria, and stem cells are examples of biological carriers (bionics) that are applied for drug delivery [123]. Among all of them, immune cells application is extended due to their surface membrane proteins which enable immune cell interactions with cancer cells [124]. To make that empirical, it is necessary to extract cell membrane, prepare the core and shell-core which need some steps to be taken including isolation of cell membrane thereafter fusion to core NPs. These cells membranes properties make bionics useful to escape from immune surveillance as well as targeted and deep penetration into target cells [125]. Bionic can also be served as a vector for drug delivery and controlled release. In particular, cell membrane camouflaged NPs have found specific interest in treatment of resistant cancer forms when combined with chemotherapy, photothermal therapy, photodynamic therapy (PDT), and immunotherapy. In the same time they can also be applied for imaging-guided detection and therapy [126]. In this line, cancer cell macrophage-membrane camouflaged nanoparticles are developed for imaging-guided photothermal therapy of resistant CRC. Persistent luminescence nanoparticles (PLNPs) contained Zn1.25Ga1.5Ge0.25O4:Cr3 + , Yb3 + , Er3 + (ZGGO) as traceable center and were coated with mesoporous silica (ZGGO@SiO2) NPs to load photothermal fluorescent dye IR825 and irinotecan. Further, a cancer cell–macrophage hybrid membrane was wrapped around this complex. Besides posseting advantages of bionics, including tumor homologous adhesion ability, superior immune escape ability, and targeted accumulation in tumor site, as an example of theranostic platforms (discussed in the next section), the developed biomimetic drug delivery system was capable of trace, diagnosis and treatment of CT-26 tumors in mice (Fig. 6A) [127]. Moreover, to improve targeting ability, combination of RBC membrane through functionalizing with tumor-specific markers is implemented. In one study, RBC membrane PGLA-coated with gambogic acid (GA) as antitumor drug and modified with recombinant dual surface protein, anti-EGFR-iRGD, as EGFR single domain antibody and tumor penetrating peptide, respectively was developed. Results showed higher antitumor effect of iE–RBCm–GA/PLGA NPs compared to RBCm–PLGA NPs and also promoted targeting efficacy and enhanced cytotoxicity in colorectal cancer cells [128].

Juglone is a natural compound with antimicrobial and antitumor properties, however it has poor solubility to be used as drug for CRC. Qian et al. developed IRGD- modified RBC NPs for Juglone and oxaliplatin to enable simultaneous delivery to HCT-116 cells. Results indicated better uptake of NPs by tumor cells, low cell viability and promoted efficient eradication of human CRC-xenografted tumors [129]. Despite extended benefits of RBC membrane derived NPs, deficiency of membrane functionalization still require further investigation. Regarding this issue, “disassembly-reassembly" technology has been studied where RBC membrane was first isolated. And endogenous lipids and proteins were excluded. After that, separated lipids were utilized to encapsulate IR780 dye. Following, original membrane protein were added to form IR780-RBC nanoparticles. This platform provided efficient photothermal therapy of CRC, as it provided higher toxicity, stability and a promising approach to afford targeted delivery with no need for functionalization of cell membrane camouflaged nanoparticles (Fig. 6B) [130]. Finally, MSC membranes coated superparamagnetic iron oxide (SPIO) NPs is used for targeted delivery of doxorubicin to treat colon cancer in MC38 tumor-bearing C57BL/6 mice [131].

#### Theranostics vectors

As practical aspects of personalized medicine, theranostics is a compilation of diagnosis and site specific therapy and disease monitoring all using a single system [132]. Different NPs are available for theranostics application including iron oxide NPs, gold NPs, quantum dots (QD), bioinspired agents like proteases, lipoproteins, viral and cellular vesicles [133]. These nanovectors can be injected to body by intravenous or local administration which is advanced by optical devices [134]. To overcome tumor hypoxia, macrophage-based carriers are used for dual delivery of nitric oxide (NO) prodrugs (photoNORMs) and Nd-UCNPs as a theranostics platform capable of providing temporal, spatial and concentration control upon different doses of NIR-light activation. These two elements, excite through NIR light and thereafter releasing of NO, hypoxia relief and deep penetration of macrophages containing theranostic microparticles into tumor spheroids can be visualized [135]. Another theranostic platform is reported composed of several components: Cetuximab (anti-EGFR antibody for targeted delivery, Ag2S quantum dots for NIR fluorescence imaging, 5-Aminolevulinic acid (ALA) induced protoporphyrin IX based for PDT and 5-fluorouracil as chemotherapeutic agent. Acting as all-in-one platform such system was applied for highly efficient combined chemo/PDT tracking (QD conjugated to Cetuximab) and therapy of EGFR overexpressing SW480 colorectal cancer cells [136]. In another preparation, TRAIL/S‑layer/graphene GD nanohybrid is used to enhance stability of tumor necrosis factor (TNF)-related apoptosis inducing ligand (TRAIL) as a main apoptosis-inducing factor and was cytotoxic (80% apoptosis) on intrinsically-TRAIL resistant HT-29 colon cancer cells [137].

Cell membrane camouflaged biomimetic nanoparticles as examples of theranostics are recently reviewed in ref [138]. Also theranostics applied for gastrointestinal cancers are fully described in ref [139].

### Computer-assisted drug delivery (CAD)

CAD system developed to provide a better drug delivery designs and address possible problems with aid of molecular modeling and simulation, data mining, artificial intelligence technique and effects of pH, temperature, salt concentration and external stimulus [20]. Three-dimensional printing (3DP) is a state-of-art novel computational system which provides controlled and sustained drug release, enabling to design complex and customize doses of drugs, printing of microneedle array, and modification of drug surface coatings. Extrusion-based core–shell printing, two-photon polymerization, fused-filament 3D printing, piezoelectric inkjet printer, fused deposition 3D printing, ink-jet printer, micro-drop inkjet 3DP, thermal inkjet printer, multi-nozzle 3D printer, and stereolithographic 3D printer are different types of 3D printing techniques which can be used in drug delivery systems [140–142]. Another important application of 3DP is assessing interaction between cyclodextrins (CD), which is a solubilizing agent and active pharmaceutical substances (API) and provide the best thermodynamic properties of CD-API systems for performing quantitative structure–property relationship (QSPR) modeling [143]. Also CD-API based system is used to evaluate solubility and antimicrobial activity of cefuroxime axetil (CA) and study can be designed by taken several steps including (I) data curation, (II) model development, (III) virtual screening, (IV) system preparation, (V) characterization (VI) dissolution tests and (VII) and antimicrobial efficacy tests [144]. Hence, to produce CD based tablets, molecular details, analyzing chemical group and molecular dynamics of CA-CD interaction need to be investigated by spectroscopy and molecular modeling [145]. Additionally, CAD approaches including a clustering algorithm and the Schrödinger software [146] machine learning (ML) techniques, ensemble learning, support vector machine, artificial neural networks and deep learning models [147] have been extended to recent pandemic concern, Covid 19 infection [148]. CAD and artificial intelligence provide new insight in colorectal cancer detection, screening and treatment especially in early stage of disease [149]. Besides that, in case of colon polyps detection artificial intelligence meets screening needs by magnifying narrow-band imaging, endocytoscopy, confocal endomicroscopy, laser-induced fluorescence spectroscopy, and magnifying chromoendoscopy [150].

### Single cell approaches for CRC treatment

Single cell technology is prospective approach in field of cancer treatment and precision medicine which can provide a profile of heterogeneities in tumors and their environment and also novel therapeutic strategies against resistance tumors can be developed [151]. Due to different response of same tumors to specific drug and heterogeneity, it seems necessary to take advantages of single cell analysis [152]. Extensive use of single cell RNA sequencing includes several steps: (I) preparation of solid tumor specimens, (II) selection of sequencing platform such as 0X Genomics Chromium, Nadia (Dolomite Bio), Illumina Bio-Rad ddSEQ Single-Cell Isolator, BD Rhapsody Single-Cell Analysis System (BD), ICELL8 Single-cell System (Takara), and Fluidigm C1, (III) analysis of single cell RNA sequencing data, including quality control, batch effect correction, normalization, cell cycle phase assignment, cell clustering, reconstructing of cell trajectory and pseudo-time, differential expression and gene set enrichment analysis and finally gene regulatory network inference [153]. Moreover single cell analysis can illustrate tumor phylogeny and their clonal evolution [154]. For instance Simulated Annealing Single-Cell inference (SASC) is a single cell sequencing based method which enable to study phylogeny of deletion mutation and cancer progression [155]. In a study, single cell RNA sequencing performed on metastatic gastric cancer patients and found diversity in microenvironment and carcinoma profile per patient. Also more specific results have revealed regarding lymph node metastasis marker and evolution driving genes [156].

Accordingly, drug delivery approaches to overcome resistant CRC can be dramatically improved if preliminary data regarding tumor status including drug sensitivity, specific mutations, epigenetic changes, immune status, heterogeneity, etc. are provided by single-cell approaches, allowing for CRC precision medicine. For one, with regard to immunotherapy resistant CRC, single cell derived colorectal organoid analysis revealed HLA peptide presentation profile and its differences among patients or even in one patient. Thus such finding can contribute to better treatment design [157]. Likewise, single cell analysis of normal colon and CRC tissue reveled WNT-independent MAPK activity in CRC as a key driver of tumor cell plasticity [158]. Furthermore, as CRC display a high degree of tumor heterogeneity, recent single-cell RNA sequencing analysis revealed heterogeneity in gene regulatory networks and identified CRC critical regulators such as transcription factor ERG [159]. In another recent work, single cell approach characterized different populations of infiltrating T cells in colon (CD8 + TN cells) and rectal cancer (CD8 + IEL cells) [160]. Very recently, single-cell and spatial analysis has revealed interaction of SPP1 + macrophages and FAP + fibroblasts in CRC. All of these examples pave the path for realizing CRC heterogeneity and thus can help design of more efficient NTDDs to overcome CRC heterogeneity and thus resistant disease.

## Conclusion

CRC as one of the pioneer diseases in mortality worldwide still face challenges for treatment. Stage at diagnosis has significant effect on mortality rates, such that diagnosing in stage IV has no chance to be cured and almost every patient who detected in stage I will be healed. Diet, gut microbiome, obesity, smoking, alcohol, chromosomal instability and CPG methylation pathway are factors that predisposed individuals to CRC. It is highly suggested to people who are under risk of CRC to undergo screening test such as fecal test, sigmoidoscopy, colonoscopy and CT colonoscopy per intervals [161].

One of the important obstacles in the way of CRC treatment is MDR which is induced by several factors including drug transporter efflux, signaling pathways, different genes mutation, EMT, tumor microenvironment features (e.g. hypoxia), CSCs, and inter- and intra-tumor heterogeneity involved in progression and resistance of CRC [5–7, 9, 12] Another contribution is CAF as most abundant and important components of CRC microenvironment. CAFs diversity is high and several extracellular, intracellular and surface markers can be defined with αSMA^Low^ CAF-A and αSMA^High^ CAF-B as two CRC distinct CAFs. CAF provide growth factors and inflammatory ligand for cancer cells. Nevertheless, anti-CAFs can be introduced as an effective approach to reshape CRC microenvironment for improved treatment. Meanwhile identifying CAFs characteristics and studding their heterogeneity provide better insight to design therapeutic strategies [162]. To overcome these obstacles, nanomedicine and targeted therapy stepped in to cancer therapy arena. These nanodrugs can impact on tumor microenvironment, DNA repair system, CSCs, cellular signaling pathways, vascular endothelial growth factors and miRNAs [163]. Thus, various nano drug delivery systems including exosomes, liposomes, hydrogels, bionics and theranostics were developed and some of them in particular liposomal drug formulation paved the way to CRC clinical trials (Table1).

**Table 1:** Summary of recent nano-based drug delivery clinical trials for colorectal cancer

Type of NP	Phase	Region	Clinical Trial NO
Bevacizumab and temsirolimus plus liposomal doxorubicin	I	United States	NCT00761644
Magnetic particle-ICG	II	United States	NCT05092750
Nano carbon and ICG	III	China	NCT04759820
TKM 080301 (lipid nanoparticles containing siRNA against the PLK1 gene product)	I	United States	NCT01437007
Liposomal Irinotecan, Fluorouracil, Leucovorin Calcium, and Rucaparib	II	United States	NCT03337087
NPs with camptothecin + CAP + RT	I/II	United States	NCT02010567
Liposomal SN-38	II	United States	NCT00311610
Liposomal floxuridine and irinotecan HCl	II	United states	NCT00361842
Liposomal PEP02 or Irinotecan in Combination With Leucovorin and 5-Fluorouracil	II	France	NCT01375816
Thermodox (Temperature-sensitive liposomes for DOX)	II	United States	NCT01464593
MM-398 (Liposomal irinotecan)	I	France	NCT02640365
Trifluridine/Tipiracil (TAS102) in Combination With Nanoliposomal Irinotecan (NAL-IRI)	I/II	United States	NCT03368963
Liposomal aroplatin	II	United States	NCT00043199
PEGylated camptothecin formulation	II	United States	NCT00931840
PEGylated recombinant human granulocyte colony stimulating factor	IV	Not provided	NCT02805166
Arginine deiminase PEGylated plus FOLFOX	I/II	United States	NCT02102022
NKTR-102/ IRI (pegylated irinotecan)	II	United States	NCT00856375
Combination of polyclonal antibody, nanoliposomal Irinotecan, 5-FU, and leucovorin	I/II	Not provided	NCT02785068
Silica NPs(Fluorescent cRGDY-PEG-Cy5.5-C dots)	I/II	United States	NCT02106598
Polymeric NPs + cetuximab + somatostatin analogue	I	Egypt, Saudi Arabia	NCT03774680

NPs should be designed based on certain principles. If the size of NPs is so small, they will be eliminated via renal infiltration, on the other hand, large NPs will not be appropriate for intravenous injection due to elimination by protein corona and phagocytic system. Also shape of NPs is important either, as spherical ones are the best for diffusion. In case of surface properties charge, roughness, and targeting moieties are important [134]. Due to significance of CRC therapy diverse efforts have been made to develop high throughput technology and methods for diagnosis, screening and treatment. For instance, liquid biopsy is prospective procedure which can provide valuable information about malignancy through rendering CTCs, ctDNA and miRNA accessible [164]. Another future prospective which extended widely in field of cancer is single cell approach. It can provide better understanding of single tumor cells including their microenvironment, cellular signaling and response. Although this approach still faces several challenges for example single cell collection needs high experimental practices due to large amount of required cells which is necessary for study. Next challenge can be described as quality control if not be considered may interrupt the results. Costly equipment is another problem in this way [165]. In conclusion, advanced drug delivery platforms, in particular theranostic ones, can be applied for precision CRC therapy in the future, as they will benefit from combined work of material, computer and biomedical scientists to design tumor-feature adoptable all-in-one drug delivery systems.

## Data Availability

Not applicable.

## Abbreviations

CRC: Colorectal cancer
MDR: Multiple drug resistance
DE: Tumor derived exosomes
CSCs: Cancer stem cells
CTCs: Circulating tumor cells
ABC transporters: ATP-binding cassette transporters
MRP1: Multidrug resistance-associated protein
BCRP: Breast Cancer
